# Driver Monitoring for a Driver-Centered Design and Assessment of a Merging Assistance System Based on V2V Communications

**DOI:** 10.3390/s20195582

**Published:** 2020-09-29

**Authors:** Sofia Sánchez–Mateo, Elisa Pérez–Moreno, Felipe Jiménez

**Affiliations:** 1 University Institute for Automobile Research (INSIA), Universidad Politécnica de Madrid (UPM), 28031 Madrid, Spain; sofia.sanchez@upm.es; 2 Psychology Faculty, Universidad Complutense de Madrid, Campus de Somosaguas, Pozuelo de Alarcón, 28223 Madrid, Spain; elisaperez@psi.ucm.es

**Keywords:** driver monitoring, automated vehicles, connected vehicles, merging maneuver, human–machine interface

## Abstract

Merging is one of the most critical scenarios that can be found in road transport. In this maneuver, the driver is subjected to a high mental load due to the large amount of information he handles, while making decisions becomes a crucial issue for their safety and those in adjacent vehicles. In previous works, it was studied how the merging maneuver affected the cognitive load required for driving by means of an eye tracking system, justifying the proposal of a driver assistance system for the merging maneuver on highways. This paper presents a merging assistance system based on communications between vehicles, which allows vehicles to share internal variables of position and speed and is implemented on a mobile device located inside the vehicle. The system algorithm decides where and when the vehicle can start the merging maneuver in safe conditions and provides the appropriate information to the driver. Parameters and driving simulator tests are used for the interface definition to develop the less intrusive and demanding one. Afterward, the system prototype was installed in a real passenger car and tests in real scenarios were conducted with several drivers to assess usability and mental load. Comparisons among alternative solutions are shown and effectiveness is assessed.

## 1. Introduction

In recent years, studies have focused on the design and development of Advanced Driver Assistance Systems (ADAS) because human error is considered to be a major factor in traffic accidents, estimated to be 94% compared to other causes such as vehicles, the environment, and unknown critical reasons [1]. Some estimates [2] ensure that road accidents and fatalities would be reduced by up to 90% thanks to assistance systems. While it is true that many of these technologies have made driving safer and more comfortable, reducing road accidents [3], there are many complex scenarios in which risk situations can happen that have not been covered yet. Situations, such as merging, lane departure, and roundabouts, require a very manual and intuitive control for the driver, where there is also a lack of knowledge of the intentions and maneuvers of other vehicles and where the same situation will never occur due to the driving style of each driver.

This paper proposes a system for providing information to the driver in merging maneuvers, which is a safe–critical situation. A driver assistance system is proposed, based on the knowledge of other vehicle positions using vehicular communications. Developing an appropriate interface is a key issue to obtain an effective system, so this paper deals with the system and interface design, evaluating both functionality and usability.

## 2. State-of-the-Art

Merging is one of the most problematic scenarios in road transport. Merging situations suppose a high attentional load since the driver must constantly recalculate and update information depending on the road characteristics and the position and relative speed of other vehicles that circulate on the main road. In addition, it means facing a task that requires high levels of concentration in a relatively short time interval, in which a failure in the maneuver can have fatal consequences. The task becomes even more complex in situations of low visibility, darkness, adverse weather conditions, denser traffic, or in situations where the vehicle’s speed is inappropriate concerning other vehicles or the road type.

The merging situation has been strongly studied among several authors. The effect of merging into traffic flow has been a well-studied topic for a long time [4,5]. Studies such as [6] conclude that the accident risk for trucks on merging ramps can vary depending on the type of ramp, the location of the conflict, and the accident type. According to the data, loop ramps have a higher accident rate than diamond ramps or directional ramps, particularly in rollovers. Another decisive factor in merging maneuvers is the time it takes the driver to perform it. This effect can be seen in [7], where the time taken to perform a merging maneuver between young people and elderly drivers was analyzed. As a conclusion, it was observed that with advanced age there is a reduction in the maneuver speed and in the information processing capacity, which is compensated by a lower speed on the on-ramp and which may imply a riskier maneuver in real traffic.

Other examples of this are [8] who studied the conditions where vehicles collide in lane changing and merging situations; reference [9] addressed the problem of connected and automated vehicles (CAVs) on access ramps to roadways, improving traffic flow and reducing fuel consumption as well as the duration of the journey; and [10] analyzed the environment of connected vehicles by means of wireless communication from Vehicle-to-Infrastructure (V2I).

In addition to those mentioned, in recent years several studies have been carried out on the subject, but all in the field of simulation [11,12,13,14]. The Multi-Driver merging simulation is well analyzed in [15], which consists of several driving simulators connected to each other. This study compares an ADAS cooperative system for merging situations in two conditions, Single-Driver simulation and Multi-Driver simulation. In the second one, all drivers are warned of the maneuver that the ego vehicle is going to carry out. The emotional response of the drivers is also presented, concluding that there are more anger and difficulty in the case of merging without an aid interface in heavy traffic than merging in scenarios with large gaps or merging with the interface. In [16], multiple control merging simulations are also discussed, concluding that simulations that include several human participants and surrounded traffic are more realistic than those of a single driver.

However, there are also studies that carry out their experiments on real roads, such as the case of [17], who carried out real driving tests with 10 subjects, and the effect of high traffic density on the state of the driver’s eye was analyzed. The results show a longer gaze duration in dense traffic situations, and the results support the need of a driving assistant that can automatically identify gaps and accelerate or decelerate the car accordingly or provide suggestions to the driver to do so. Through connected vehicle technology and a decentralized algorithm, Salman [18] developed a freeway merging system, which provides a visual warning on a Google map on a smartphone via Bluetooth, tested in real life on an interstate highway in an uncontrolled environment. This system only indicates the need to accelerate, brake, or enter a gap, using an algorithm based on communications between three vehicles: two driving along the freeway and the third one trying to merge into the main lane.

Thus, the tendency is to develop an assistance system for merging, which will improve traffic and reduce the driver’s mental load during the maneuver. In order to know the cognitive mechanisms that exist in this situation, one of the most common alternatives is using measures of an eye tracking system, analyzing ocular movements and extracting information from parameters such as pupil diameter and duration of gaze fixations [19,20]. Studies have shown that merging situations demand a higher cognitive load than usual [21,22,23].

Considering this state-of-the-art, an application for merging assistance was developed in this work, based on Vehicle-to-Vehicle (V2V) communications, and parameters of the cognitive load were studied by means of an eye tracking system. This system provides the driver information to take accurate decisions on how to perform the maneuver. The system proposes the speed the vehicle should reach based on road and traffic conditions. The developed application was tested in a simulator and validated in real tests using V2V communications, compensating the shortcomings found in previous studies, such as the lack of tests in a real environment [15,17,18] and the lack of an interface systematic design, as well as the lack of evaluation of usability and acceptability by the user, which is quite common in human–machine interface (HMI) prototypes. Due to the relevance for safety of these maneuvers and the high cognitive load that could generate to drivers, this work tries to select the most convenient interface and to corroborate its usability and usefulness as well as overall driver satisfaction.

This paper has as its starting point previous research on the same subject [21,22,23], in which driver attention on safety–critical scenarios have been tested and assessed. Section 3 shows a preliminary justification of the need of assistance systems in the safe–critical scenario of merging lanes. Section 4 explains the assistance system definition, based on V2V communications, the control architecture, and the algorithm that controls it. Section 5 corresponds to the design of the HMI and the tests carried out on the simulator for the choice of the interface to be integrated into the assistance application, considering objective and subjective measures (Satisfaction, Usefulness, Usability, and Mental Load) obtained from a sample of drivers. Section 6 shows the real tests carried out with the merging assistance system so its impact could be assessed. Finally, Section 7 provides the main conclusions.

## 3. Justification

A survey on behavior and sensations in merging carried out during the experiments corroborates the facts that support the previous statements. This survey served both to know the stress level perceived by the drivers and the style and behavior of these in merging situations into another road. Being a short online survey of 33 questions, it was possible to obtain a wide variety of profiles. A total of 99 participants responded to the survey, of which 53 were men and 46 women, aged between 18 and 72 years (mean = 37.68 and SD = 11.02), with a driving experience of up to 48 years (mean = 17.07 and SD = 11). About 90% of them regularly drive a car. There was a similar number of respondents at different levels of distance traveled per year (less than 5000, between 5000 and 10,000, between 10,000 and 20,000, and more than 20,000 km/year).

The correlation between the different results of the survey was analyzed, obtaining the dimensionless value r. The value r oscillates between −1 and +1, being the signs for the positive or negative linear correlation. Table 1 summarizes the most noteworthy correlations.

The main results obtained conclude that the variable years of driving experience has a negative linear relationship with the stress experienced in different merging scenarios. In the same sense, it correlates with the driver’s age. This suggests that there is a tendency for drivers with more driving experience, as well as older drivers, to have lower levels of perceived stress in merging situations and for those with less experience, as well as younger drivers, to have higher perceived stress. Both variables (experience and age) are closely linked, so the experience variable is the most relevant in this context. There is a tendency for drivers with higher levels of stress to wait until the end of the lane to start braking, to be riskier, and to start merging earlier if they notice vehicles behind them start with the maneuver first and use the horn on the highway more frequently. On the other hand, drivers are usually less risky if they carry passengers in their vehicles.

In any case, most drivers report that they rarely, or only sometimes, feel stress when they merge (approximately 70–80%); these stress levels are higher when the driver arrives without sufficient speed at the end of the lane, visibility conditions are poor, or they find a truck or bus in the lane where they wish to merge (50–60% recognize feeling a medium or high level of stress). On the other hand, approximately half of the participants recognize that if you reach merging with enough speed, the rest of the vehicles ease the maneuver. Only 15% of drivers stop before the acceleration lane signal if they cannot merge, 35% stop after the signal, and 50% stop at the end of the acceleration lane. Approximately half of the drivers do not stop at the beginning of the lane even if they have a short acceleration lane and do not see the exit clearly. However, the majority of interviewees (more than 80%) facilitate the merge of other vehicles onto the road on which they travel when conditions permit. Mainly, drivers when merging are looking at the vehicles on the road, the vehicle in front, and the length of the acceleration lane.

With these results, we can conclude that the stress variable is associated with different undesirable behaviors of drivers on the road when they merge; for example, waiting until the end of the lane to brake when they cannot merge or being riskier when other drivers behind the driver want to merge before them. This relationship is probably not causal, but it seems appropriate to analyze how far a system to assist merging can improve and facilitate these maneuvers.

That is why a merging assistance system is proposed, which the driver can consult while performing the maneuver and provides information on the vehicles on the road where it is intended to merge. The system should advise in terms of speed how much to accelerate or decelerate to perform the maneuver safely, either in front or behind the vehicle.

## 4. Assistance System Definition

The purpose of the assistance system is to help drivers while merging, providing them information about the right moment to enter the main road or how to perform this maneuver in terms of speed control (accelerating or decelerating, depending on the situation). It has been implemented in a smartphone that serves as the driver interface and implements the algorithm that handles the information from V2V communications and from a digital map. Although using a smartphone while driving is illegal in many countries, it must be noted that, in this system, this device only plays the role of the human–machine interface and cannot be manipulated during the driving task. Any previous configuration of the system must be introduced before starting the trip. This section provides the description of the technological parts of the assistance system.

### 4.1. Hardware and Information Sources

Vehicle-to-X (V2X) communications allow information exchange between vehicles, so the environment is converted into a cooperative system, which gives the vehicle a wider horizon of information, so that it can anticipate the decision making. Some C-ITS experiments, such as [24,25], employed vehicle-to-infrastructure (V2I) communications to display road information panels to the driver inside the vehicle by means of a human–machine interface (HMI). Several experiments also used vehicle-to-vehicle (V2V) communications for the exchange of information between vehicles [26,27]. Other solutions can be seen in [28,29], where V2I communication can be understood as a network of crossroads, traffic and construction signs, speed limits, and traffic signal information to automated vehicles. Similarly, V2V communication allows vehicles sharing their own information [30,31]. By integrating V2V and V2I communication with automated vehicle systems, an efficient “cooperative driving” network can be developed [28,32].

For these purposes, the research team has developed communication modules that could be placed both in the vehicles and the infrastructure [33,34,35,36]. The Decentralized Environmental Notification Messages (DENM) provided by a G5 On-Board Unit (OBU) installed in the vehicle are shared by all vehicles in a certain area and, optionally, these communication modules could be connected to Road Side Units (RSU) linked to the Traffic Management Centers of the Directorate General of Traffic (DGT TMC). Furthermore, the OBU incorporates a Global Navigation Satellite System (GNSS) NV08C-CSM chipset with an update frequency of 5 Hz. These OBUs are installed in vehicles in order to provide the information of absolute positioning and speed to other vehicles in the surroundings. Vehicles in a merging lane use this information as described in the following subsection.

Furthermore, a digital map with previously included information of the merging lanes is also used and specific map-matching algorithms are used for vehicle positioning on the map [37].

### 4.2. Algorithm

**Figure 1:**
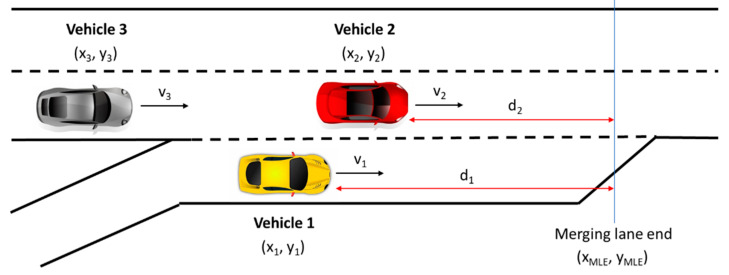
Vehicles in the merging lane (Vehicle 1) and in the main road (Vehicles 2 and 3), and the variables involved in the algorithm.

The merging algorithm indicates the need to accelerate, decelerate, or enter the main road and is more intuitive and simpler than the one presented in [18], for example. Figure 1 illustrates the position of the vehicles involved in an instant in which the algorithm is trying to decide how Vehicle 1 could enter the main road, considering that *d_i_* is the distance between vehicle *i* and the end of the merging lane, and *v_i_* is the speed of vehicle *i*. The algorithm is implemented in a mobile application with an iOS system.

The algorithm output is based on previous studies in which an intelligent speed adaptation (ISA) system was developed [38]. That system informed the driver about the speed to be acquired when reaching a road section, and suggested the driver, in qualitative terms, how much to accelerate or decelerate when approaching that section. In the case of the merging assistance system, the driver should receive similar information of how to adapt his speed to merge onto the main road in safe conditions. The main premises are as follows:

1. The safety margin between vehicles must not be less than two seconds because of previous studies on driver reaction time;
2. The maximum speed of the road in the acceleration lane must not be exceeded in any case;
3. The assumed acceleration and deceleration limits are 2 m/s^2^ and 4 m/s^2^, respectively.

The reason for defining a safety margin in terms of time is because higher speeds require larger distances to brake. Several studies maintain that a driver reacts, in the worst case, with a reaction time of 1.5 s to a surprise event, as an object that moves suddenly on the driver’s route [39]. However, other studies found larger reaction times [40]. This is why, in a conservative way, a two seconds reaction time is chosen. The acceleration and deceleration levels have been stated considering state-of-the-art values. In [41], a collision warning system is implemented, where three levels of sensitivity are established at the driver’s choice. The value of 4 m/s^2^ for deceleration has been taken, corresponding to the high sensitivity level to maintain greater distances. For the value of acceleration, Prestl [42] considered the range of accelerations for an intelligent system of this type must be between −2 m/s^2^ and 1 m/s^2^ to avoid problems in the traffic flow in case of an inappropriate decision.

The control algorithm calculates the constant acceleration/deceleration that is required for Vehicle 1 in the acceleration lane in order to merge onto the main road where Vehicle 2 is moving at a constant speed. This acceleration calculation as a function of time *t*, given by Equation (1), is executed continuously so the speeds of both vehicles are constantly updated.

(1) $$a_{1}\left(t\right)\;=\;\frac{d_{1}-v_{1}\cdot t}{0.5\cdot t^{2}}\;$$

**Figure 2:**
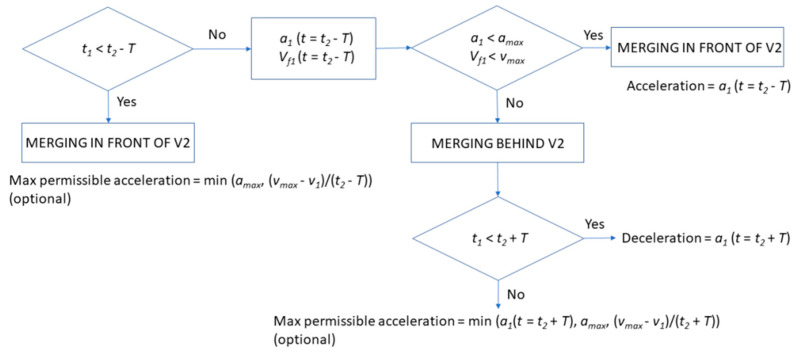
Flowchart of the decision algorithm.

The decision tree shown in Figure 2 starts considering that both vehicles maintain their speeds and the variable that determines the required action is the relationship between how long they take to reach the end of the merging lane (*t*_1_ and *t*_2_), given by Equation (2).

(2) $$t_{i}\;=\;\frac{d_{i}}{v_{i}}\;$$

In case Vehicle 1 reaches that point earlier than Vehicle 2 (considering the safety margin), no action is required for merging behind Vehicle 2 (and Vehicle 1 could even accelerate). In case the first condition is not fulfilled, the algorithm decides whether merging is executed in front of or behind Vehicle 2. The first scenario is possible if the maximum acceleration *a_max_* and maximum speed *v_max_* are not exceeded, to guarantee the safety margin; estimating the final speed of Vehicle 1 is done as per Equation (3).

(3) $$v_{f1}\left(t\right)\;=\;a_{1}*t+v_{1}$$

Otherwise, merging must be executed behind Vehicle 2. In this case, if the difference between *t*_1_ and *t*_2_ is smaller than the safety margin *T*, a deceleration is required; but, if not, Vehicle 1 could accelerate up to a maximum level. It must be noticed that, in case the algorithm decides that merging in front of Vehicle 2 is not possible, but merging behind it is feasible, the same flow chart is repeated with the vehicle behind Vehicle 2 in its lane (Vehicle 3), to verify that merging in front of it is safe enough. This process is repeated until a safe maneuver is found.

The control algorithm used has a low computational load and it works correctly in real time, which is an advantage over other similar studies.

## 5. Interface Design

Human–machine interfaces must be designed in the most ergonomic way, both physically and mentally, to avoid being a distraction for the driver, but in many cases, when designing an ADAS, usability and user satisfaction are not usually evaluated and this fact could lead to warnings, misunderstanding, confidence losses, or a usefulness perception of the system. Furthermore, as the scenario involves a high attentional load, this HMI assessment is even more crucial. Some authors, such as Biondi [43], have developed a scale to assess the interface of several different assistance systems.

As premises for this study and for the construction and subsequent evaluation of the different proposed interfaces, the Commission Recommendations of the European Communities of 26 May 2008, relating to safe and efficient in-vehicle information and communication systems, were followed [44]. In [45] are summarized some of the desirable features on the devices in the vehicles. For example, no average glance duration should be greater than 1.2 s and the device must not affect the vehicle control, neither to driver workload nor its situational awareness, attracting the person’s attention only if necessary. The importance that the device need not only be safe and efficient but also accepted by the user, in other words, that it is perceived as useful and pleasant and that its use does not require any specific training, is pointed out in [46].

Furthermore, the designs chosen are based on the research group’s own experience in designing and validating interfaces for safe driving on mobile devices in previous applications [38,47].

### 5.1. Interface Location

In a previous study of the authors [22], two different scenarios of traffic density were shown, one involving a high attentional load for the driver (HAL) and another one of low attentional load (LAL). After this study, in [21,23], a Wilcoxon signed-rank test to paired-samples data was carried out for pupil diameter in merging situations and in baseline situations, whose values for the right eye were Z = −2.38 and *p* = 0.016 and for the left eye were Z = −2.1, *p* = 0.036. These values represent that there are statistically significant differences between both situations. The fixations were also analyzed using heat maps, with a remarkably hot zone common to both rear mirrors. When the subjects performed the merging maneuver, a large percentage of their fixations were located on the upper-inner part of the mirror in more than half of the tests conducted. This conclusion serves as a precedent for the proposal of the merging assistance system that is placed at the bottom of the vehicle A-pillar, in order to support the driver during the maneuver.

### 5.2. HMI Design Proposals

**Figure 3:**
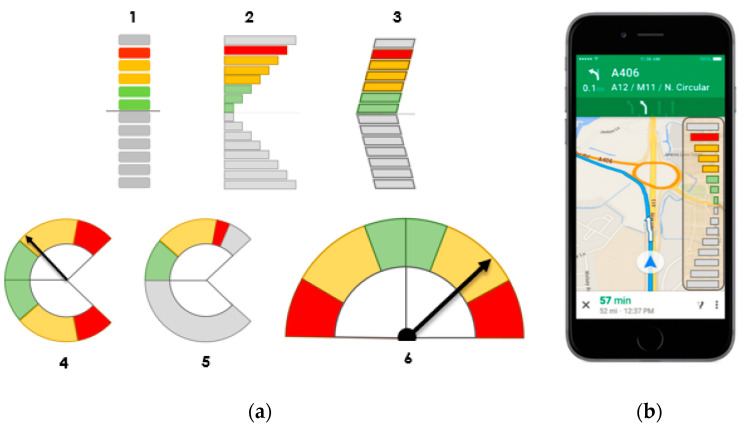
(**a**) Proposed interface alternative designs. (**b**) Example of the implementation in the smartphone of one of the alternatives.

The proposed interfaces are shown below. Some of them are derived from the basic design and others are based on the field of aeronautics, in which variometers indicate to the pilot whether it is ascending or descending into an air mass. In summary, the proposed designs are based on a set of bars or circumferential segments, as can be seen in Figure 3.

From the proposed interfaces, a team of experts belonging to the Department of Psychology of the Complutense University of Madrid finally chose three, Interfaces 1, 3, and 6, due to their operability and ease of understanding. Interface 1 was chosen as the simplest of those of that typology; Interface 3 is chosen because, in addition to the information provided by Interface 1 and the similarity with Interface 2, it adds the direction, which can point the side where to merge; and, finally, despite its similarity to Interfaces 4 and 5, Interface 6 was chosen due to its similarity to the speedometer of a vehicle control panel, to which drivers are used to and implies a completely different concept compared to the previous ones.

### 5.3. Interface Selection in Driving Simulator

In order to know the acceptability of these HMIs, both technically and socially, and to obtain a robust validation, several tests have been proposed in a driving simulator. The aim of these tests is to find out which interface design is less distracting for the driver and has more acceptance by the participants. The chosen interface would be implemented in the final system for the real driving tests.

#### 5.3.1. Participants

Twenty-three participants (N = 23, 12 females) participated in this study, aged between 18 and 53 years (M = 28, SD = 9.4). Their average driving experience was 8.4 years (SD = 9.4).

#### 5.3.2. Instrumentation and Data Acquired

Tobii Pro Glasses 2, a state-of-the-art eye tracking device consisting of glasses and a software controller connected to the eye tracker via an HDMI cable, were used for monitoring the driver activity. The glasses include two IR sensors and two cameras in each eye, which allows acquiring gaze and pupil data. In addition, a camera placed in the center and facing to the front recorded the video of the scene, in such a way that the software returns an image of the visual field of the driver and superimposes the point the driver is looking at in real time. The tests took place in a driving simulator in the laboratory. The eye data were recorded at 50 Hz with a spatial accuracy of 0.63° at 1.5 m. The transmission protocol is the one provided by Tobii [48], which operates on UDP. To analyze ocular data, Tobii Pro Lab software was used. In addition, data were collected of the driver’s actions in the simulator. To evaluate the effect of the amount of light on the pupil, with and without an interface, a Digital Illuminance Meter model ISO-TECH 1332 A was used.

#### 5.3.3. Procedure

The experiment was carried out in a Faraday cabin. Participants sat in front of a projector screen where three driving simulations of 5 min each were projected and in which they carried out the task of simulated driving. In each simulation, a different interface design was incorporated, which was presented counterbalanced by the participants. Before starting the experiment, participants were instructed to drive naturally and follow the interface indications. In addition, each of them configured the operation of the system as they understood as more intuitive.

Regarding the data analysis, the design was within-subject. Each participant performed three experimental situations, one for each type of interface. For each one, the following variables and indexes were measured: (A) system acceptance (Satisfaction, Usefulness, and Usability); (B) mental load (through a subjective measure, Rating Scale Mental Effort (RSME), and a physiological measure, the Pupillary Dilation); (C) eye measurements (total time looking at the interface and number and average duration of fixings); and (D) measurement of interface tracking (percentage of tracking of the interface according to its state in speed and direction).

After each experimental condition, where a different interface was shown, the Systems Acceptance questionnaire of [49] was answered by the participant. This questionnaire consists of nine 5-point rating-scale items, ranging from −2 to 2. These items score on two scales: Usefulness of the system and Satisfaction. In addition, the participants answered the System Usability Scale [50] and the Rating Scale Mental Effort [51]. At the end of the three driving simulations, they also answered the I-Driving Scale [52] and some questions about demographic information, as well as whether or not they would implement the application on their mobile if it was free. The average duration of each session was about twenty minutes.

Visual information is acquired during eye fixations [53]. A longer duration of the fixations in one interface versus another would indicate that the former requires a longer time to process the information and thus involves a greater distraction from the main driving task. However, in this context, the number of fixations, or the total time looking at the interface, informs of better tracking of it. In this sense, we must remember that the interface state is changing, indicating continuously the speed required to perform a safe merging. With a dynamic interface, the driver needs to analyze the current state of the interface, react, return his gaze to the road, and look again at the interface to check the new status. In an interface that works properly, more fixations but of brief duration are expected, indicating that the driver is not distracted and that it is easy to process the information presented.

For each dependent variable, a unifactorial Repeated Measures Analysis of Variance (ANOVA) in the interface design was performed. The adjustment of the Pairwise Comparisons was performed by the Bonferroni method. For each variable, three contrast conditions were considered (Interface 1, Interface 3, and Interface 6), except for the pupil diameter variable, in which the condition of the pupil diameter in the situation of driving without any interface was introduced in the analysis. In this last analysis, furthermore, a Helmert contrast was carried out, allowing the comparison between the pupil diameters when driving without the interface (baseline) versus the pupil diameter when the interface is active.

### 5.4. Results

#### 5.4.1. Measures of Satisfaction, Usefulness, and Usability

**Figure 4:**
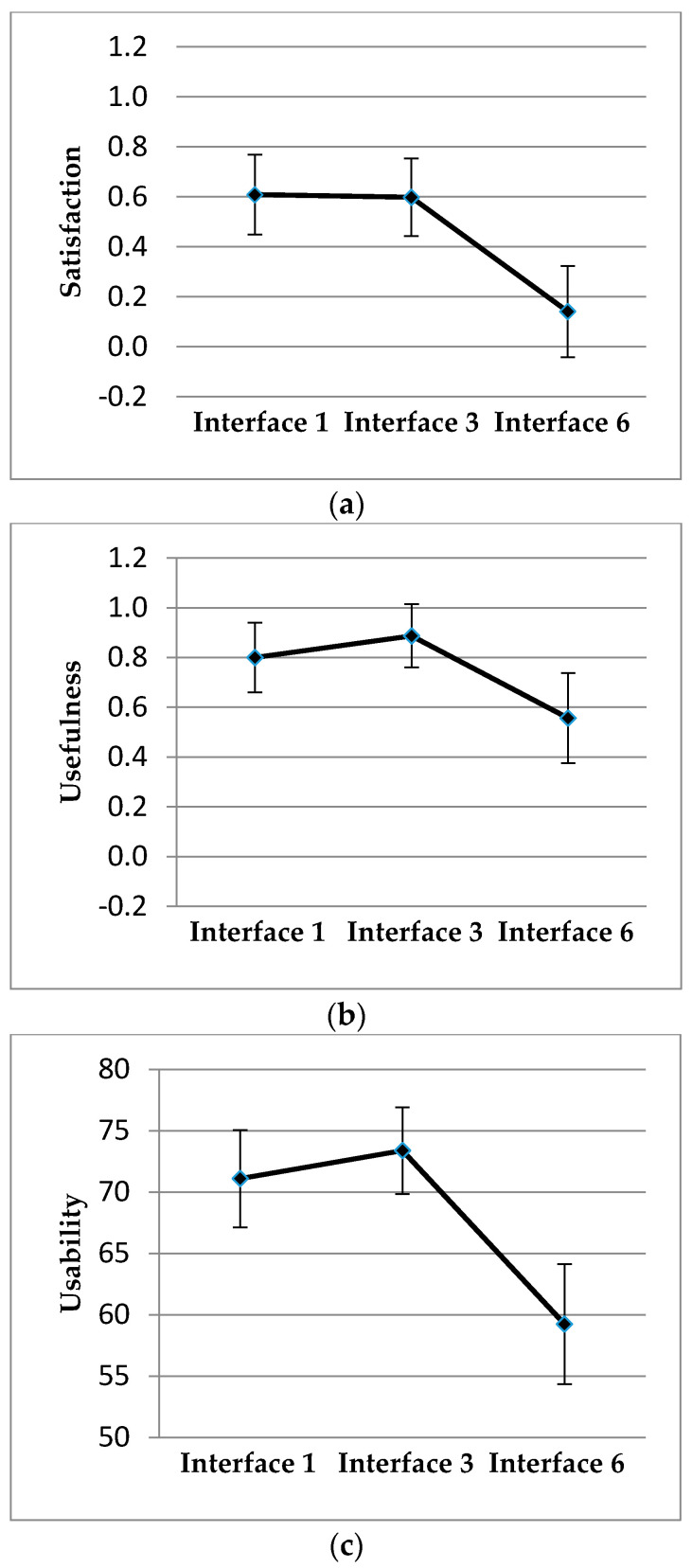
Mean and standard error of (**a**) Satisfaction; (**b**) Usefulness; (**c**) Usability.

Statistically significant differences in the evaluation of the drivers between the different types of interfaces in Satisfaction, Usefulness, and Usability were found, with F (2;44) = 7.7, *p* = 0.001, η2 = 0.26; F (2;44) = 3.53, *p* = 0.038, η2 = 0.14 y F (2;44) = 9.2, *p* < 0.001, η2 = 0.3, respectively. Interfaces 1 and 3 were evaluated as equal and more satisfactory, useful, and usable than interface 6 (Figure 4).

#### 5.4.2. Measures of Mental Load

**Figure 5:**
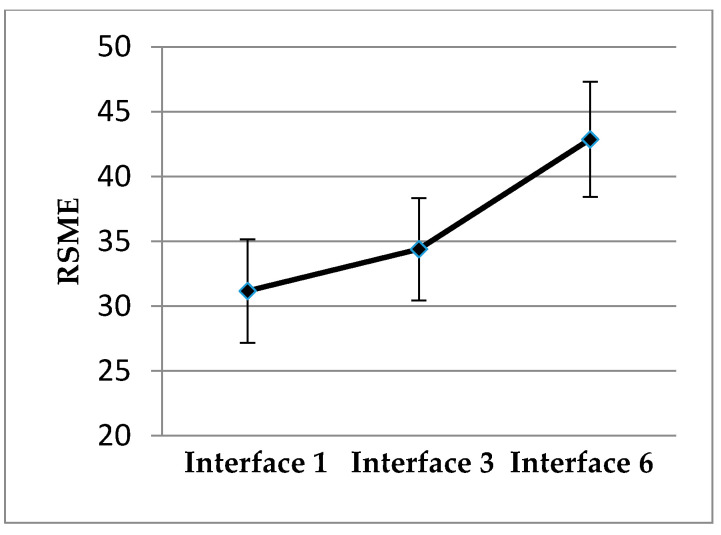
Mean and standard error of Rating Scale Mental Effort (RSME).

Regarding the results in the RSME, statistically significant differences in the mental load that requires the follow-up of the different interfaces were found, F (2;44) = 5.57, *p* = 0.007, η2 = 0.2. The drivers evaluated that Interface 6 supposes a greater effort than Interfaces 1 or 3, and no differences between these last two were found. The results can be seen in Figure 5.

**Figure 6:**
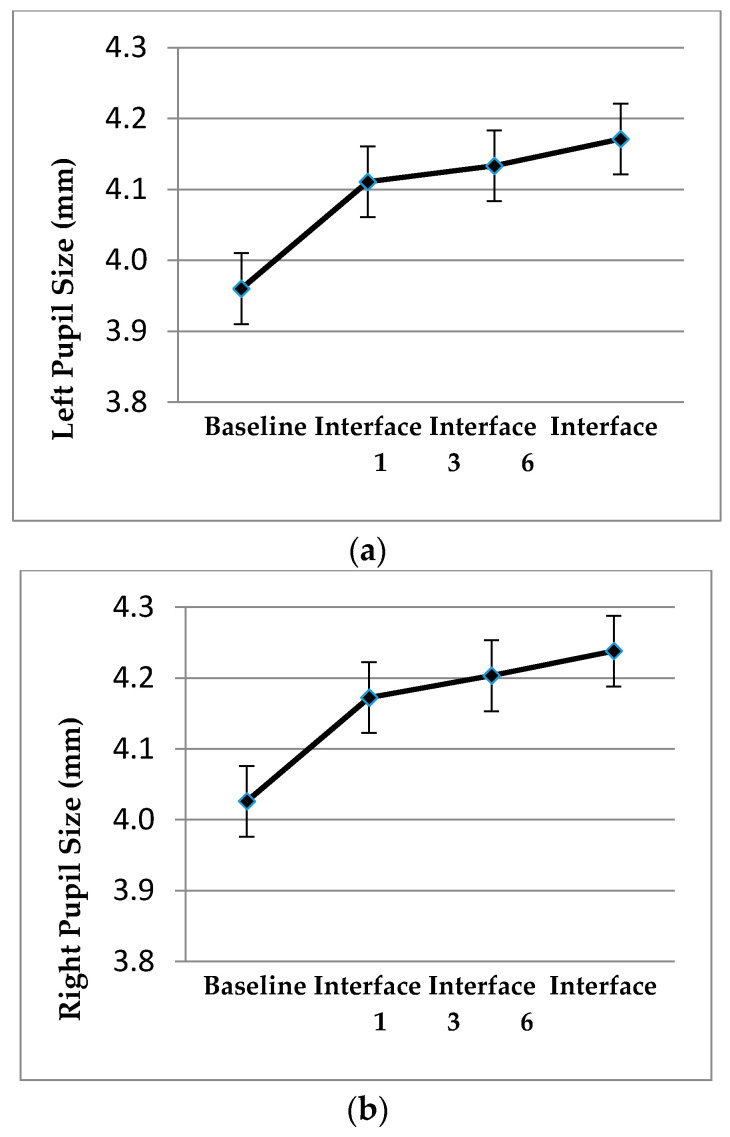
Mean and standard error of left and right pupil diameter according to the absence/presence of the interface and the interface design.

For the pupil diameter variable, a statistically significant difference between the different conditions was found, with F (3; 57) = 19.68, *p* < 0.001, η2 = 0.51 for the left pupil and F (3; 57) = 17.31, *p* < 0.001, η2 = 0.48 for the right pupil. These differences occur between driving without the interface (baseline) and when the interface is active, being clear that the pupil dilates more in the second case. However, there are no statistically significant differences between the conditions with the different interfaces. Probably, these differences are reflecting the mental load difference performing a merging situation versus driving performing any other maneuver (baseline) (Figure 6), rather than following the interface advice. It must be considered that the pupil diameter is not affected by light conditions in these tests because the conditions in the laboratory do not change.

Regarding other measures used, such as eye measurements and measurement of interface tracking, no statistically significant differences in the study depending on the interface design were found. A summary table of these measures’ analyses with the values of their mean and standard error of the mean is shown in Table 2.

### 5.5. Discussion and Interface Design Selection

According to the results obtained in terms of the eye measurements and measurement of interface tracking, it is not possible to conclude a different operation of the three evaluated interfaces. This fact is reasonable, considering that the three interfaces are the result of a previous selection.

However, there is a difference between the user acceptance of Interfaces 1 or 3 versus Interface 6, in the sense that the first two are considered more satisfactory, more usable, and more useful than Interface 6. These characteristics are fundamental when choosing or starting up a new system in vehicles. According to [49], a prerequisite for the introduction of new in-vehicle technology is the acceptance because it is unproductive to invest effort in designing and building an intelligent co-driver if the system is never switched on, or is even disabled.

Considering jointly the results for the two variables of mental load (RSME and Pupillary Diameter), it can be concluded that following Interfaces 1 and 3 is more effortless than Interface 6. Therefore, although the difference in the pupil diameters is not statistically significant, it follows the same trend as the effort evaluation. On the other hand, the pupil diameter results reveal that, when a driver is in a merging situation, the mental load is greater than in an ordinary driving situation.

Given all the results, Interface 6 is discarded to be implemented on the final system and Interface 3 is chosen for the next stage, due to the fact that this interface reports similar results as what Interface 1 provides, but provides more information.

## 6. Implementation of the Final System on a Real Vehicle

For the correct validation of the merging assistance interface, it was implemented in a mobile device placed in a vehicle to perform tests in real driving conditions in which attentional measures were analyzed recording eye movements, measures of mental load, and measures of acceptance of the system.

### 6.1. Methodology

In order to assess the suitability of the driver assistance system, the experiments from real driving were evaluated in the same conditions as in the driving simulator, by means of eye tracking and a questionnaire that summarizes aspects of usability, acceptability, and mental load, but a questionnaire on mental effort was introduced. This questionnaire is the National Aeronautics and Space Administration’s Task Load Index (NASA-TLX) [54], which is widely used and has achieved solid arguments in human factors research [55,56]. It is also used today to assess subjective workload and cognitive effort for a task projected on an HMI as in [57,58,59]. The application is installed on an iPhone device that connects with the vehicle module via WiFi.

#### 6.1.1. Participants

Thirteen participants (N = 13, 2 females) participated in this study, aged between 24 and 43 years (M = 31.53, SD = 6.35). Their average driving experience was 11.53 years (SD = 6.59).

#### 6.1.2. Instrumentation and Data Acquired

A vehicle with automatic gear shift to free the driver from the process of changing gears was equipped with the smart phone placed near the vehicle A-pillar, with the merging assistance application installed. This vehicle and the vehicles driving along the main road were equipped with OBU C-ITS G5 communication modules. The assistance application generates only visual warnings based on the speed information and the positioning collected by the GNSS on a digital map. To assess driver behavior, apart from acquiring actions on the vehicle, the same eye tracking system was used as in the previous section, Tobii Pro Glasses 2, and the same variables were analyzed.

#### 6.1.3. Procedure

The real driving tests were carried out on a stretch of the M−45 motorway near Madrid (Spain), with three signaled acceleration ramps. Before performing the real tests, drivers had to complete the first part of the NASA mental effort questionnaire, in which they subjectively evaluate the workload of a task. Then, tests were performed with the instrumented vehicle and the assistance system to help them in the merging maneuvers. The average duration of each session was approximately fifteen minutes driving and five minutes more to answer the scales.

It must be considered that these mobile devices located inside the vehicle can both aid the drivers or distract them from the main driving task [60]. In order to evaluate the functioning of the interface, various attentional measures were recorded by means of (A) measures of the assistance system acceptance; (B) measures of mental load; and (C) measures of eye movements. Different measures of system acceptance were taken for each participant at the end of the driving tests, answering the System Usability Scale [50] and the Systems Acceptance Questionnaire of [49]. In order to evaluate the mental load using the assistance system, ratings were recorded on the NASA Task Load Index [54], and on the Rating Scale Mental Effort [51], as well as the pupil diameter (during merging maneuvers with and without the assistance system).

In order to evaluate eye behavior, the following parameters were recorded: the average number and duration of fixations to the interface; the average duration of the first fixation; the number and average duration of looks at the interface (a look is defined as each time the gaze remains on the interface, with one or more fixations before looking away from it); and the average total duration of the gaze on the interface (i.e., the average total time spent looking at the interface each time it appears or the sum of the duration of the looks).

The average duration of ocular fixations is considered as a distraction measure. A greater duration of the fixations to the interface would imply a greater time to process the information. However, in this context, the number of fixations or the total time looking at the interface report a better tracking of the interface. In this sense, it should be remembered that the interface is continuously changing with the situation and this fact could involve more frequent fixations but of short duration, even with a proper interface.

Due to the small sample size, the results cannot be analyzed as in the previous section, where tests were carried out on a driving simulator. For the data analysis, a Wilcoxon signed-rank test to paired-samples data was carried out to verify whether there are statistically significant differences between the pupil dilations in both eyes during merging with and without the system. This test was considered because it is not possible to determine whether the sample comes from a population with a normal distribution. This test could be considered the equivalent of the t-test for paired-samples but operates with ranges instead of means. Some correlations between the variables are also reported using the Pearson (*p*) correlation coefficient.

### 6.2. Results

#### 6.2.1. Measures of Satisfaction, Usefulness, and Usability

Results of the mean and standard deviation of the tests carried out under real driving conditions are shown below. The results for the Measures of Satisfaction, Usefulness, and Usability are only descriptively comparable, as shown in Table 3.

#### 6.2.2. Measures of Mental Load

Mental workload measurements have been evaluated using the RSME and NASA-TLX questionnaires, which are highly used to evaluate the task load in different fields. In Table 4, results of the mean and standard error are shown.

Regarding the eye tracking measures, no statistically significant differences were found between pupil size during incorporations with or without system function, neither for the pupil of the left nor right eye (Z = −0.594, *p* = 0.552 and Z = −0.664 *p* = 0.507, respectively). These results support the hypothesis that in both situations the mental load is the same, probably due to the effort required by the merging situation. These results seem to indicate that the use of the assistance system does not imply an over-exertion for drivers (Table 5).

#### 6.2.3. Correlations between Measures

Correlations have been made between all the measurements obtained during the real driving tests, finding statistically significant correlations for some of them. The variables analyzed are shown in Table 6 and the results obtained using the Pearson’s correlation coefficient (ρ) in Table 7:

These correlations are interpreted as follows: On the one hand, when a driver is more satisfied with the system, he perceives it as more usable and mentally less demanding. In addition, the more useful the system is evaluated, the greater the number of looks at the interface, which implies greater tracking. However, the worst the assessment of the mental workload involved in the tracking the system, the higher the number of fixations to the interface, probably reflecting a greater need for information processing.

Finally, it is interesting to underline the strong association between pupil size and scores on the RSME scale for both measures of mental load, one being a physiological measure and the other a subjective measure, making the latter a valid tool to assess mental workload in real traffic situations.

### 6.3. Discussion

In the system acceptance measures, it can be observed that the average values obtained in the three scales are over half of the score, highlighting more usability and slightly less satisfaction and usefulness.

Regarding the measures of mental load, in the same way as in the measures of acceptance, the analysis should be only descriptive. The values obtained in both questionnaires are below half of the score, the result of the RSME being considerably lower than that of NASA TLX. These results indicate that users rated the interface performance as effortless, an important feature when implementing a new technology in a vehicle.

The measures obtained from the visual tracking system show that the driver assistance system for merging situations does not distract the driver [44]. The number of looks at the interface for each merging maneuver (M = 8.56; SD = 3.22) is considered low—their average duration was 492.22 milliseconds (SD = 181.62)—which is a favorable result for the desirable characteristics defined by [45]. The total time looking at the system in each merging maneuver was also short, only 4.07 s (SD = 2.31), so the results are considered satisfactory.

## 7. Conclusions

Merging maneuvers are quite safe critical and assistance systems that could help the driver to perform them in a safer and more satisfactory way. This paper presents the definition of the system from a technological point of view, but it is mainly focused on the proper HMI design in order to enhance acceptance and effectiveness, performing a proof of concept of the driver’s behavior when using it.

The merging assistance system presented in this paper is based on communications between vehicles and its interface shows the driver, in a passive way, the speed recommendations to perform the maneuver in the safest possible way. The intentions of the main road driver are clarified thanks to the decision algorithm based on the position and speed variables of both vehicles.

In this design, certain premises were followed, such as the recommendations of the Commission of the European Communities concerning safe and efficient in-vehicle information and communication systems [44], the desirable characteristics of in-vehicle devices according to [45], and the considerations mentioned in [46].

The system was validated not only in a driving simulator but also in a real driving environment, in which vehicles exchange information using V2V communication modules. Thanks to an eye-tracking system and several system acceptability surveys performed by drivers, it was possible to select a driver assistance interface for merging situations. The proposed method has proved its value in distinguishing the best interfaces without any default consideration and combining several criteria, such as satisfaction, usefulness, usability, and mental effort. The results from the driving simulator and real vehicles show that the selected interface is well accepted by the users.

Comparisons of the final system with other similar studies are not possible without replicating those developments as the test environment and conditions are different (for example, driving simulator tests and real driving tests are not directly comparable). The results presented in this paper try to show the whole design process with the drivers’ assessment at each stage, and this fact is the main difference with previous assistance systems for the same problem and the pillar for obtaining such good results in usability and low mental load. Finally, a smartphone was selected for the HMI device because it provides flexibility in the design and integration process, but future integration of the interface is intended to be within the vehicle controls or integrated in the structure of the vehicle, not as a separate device, to improve the visual conditions of the driver position. This new configuration would provide even better results because mobile devices may distract drivers more easily.

## Figures and Tables

**Table 1:** Correlation coefficient between variables.

Variables	Correlation Coefficient r	*p*-Value
**Driver’s features**	**Between driver’s features and stress experienced in merging scenarios**	
Driving experience	−0.353	<0.001
Driver’s age	−0.281	<0.005
**Action**	**Between action and drivers with high stress perceived**	
Braking at the lane end	0.271	0.007
Being riskier	0.365	<0.001
Start merging earlier	0.309	0.002
Using the horn frequently	0.225	0.025
**Situation**	**Between situation and less risky drivers**	
Carry passengers	0.359	<0.001

**Table 2:** Mean and standard error of the mean for the variables analyzed in the driving simulator.

Variable	Interface 1		Interface 3		Interface 6		*F*	*p*-Value
	Mean	SE	Mean	SE	Mean	SE		
TTL (s) *	3.78	0.42	4.71	0.61	4.11	0.51	2.54	0.09
Number of Fixations	8.03	0.56	8.09	0.47	8.11	0.59	0.01	0.98
Fixation Duration (ms)	406	25	468	40	447	34	2.04	0.15
PTS *	49%	0.005	50%	0.005	49%	0.006	0.16	0.85
PTSW*	73%	0.03	71%	0.05	74%	0.04	0.07	0.8

* Note: TTL (total time looking at the interface); PTS (percentage of time the driver follows speed notifications); PTSW (percentage of time the driver follows steering wheel notifications).

**Table 3:** Mean and standard error of Satisfaction, Usefulness and Usability.

	Satisfaction	Usefulness	Usability
**Mean**	0.3269	0.1385	65.193
**Standard Error**	0.1843	0.1623	4.7910
**Scale**	−2; +2	−2; +2	0; 100

**Table 4:** Mean and standard error of RSME and NASA-TLX.

	RSME	NASA-TLX
**Mean**	37.6923	43.7179
**Standard Error**	4.6207	3.6384
**Scale**	0; 150	0; 100

**Table 5:** Mean and standard error of left and right pupil diameter during merging with and without the assistance system.

	Left Pupil Diameter		Right Pupil Diameter	
	With ADAS	Without ADAS	With ADAS	Without ADAS
**Mean**	2.2754	2.2780	2.2915	2.3070
**Standard Error**	0.05	0.05	0.05	0.05

**Table 6:** Mean and standard error of the mean for variables analyzed under real driving conditions.

Measures	Mean	Standard Deviation
Usability (0–100)	65.19	17.27
Usefulness (−2–+2)	0.14	0.58
Satisfaction (−2–+2)	0.33	0.66
Left pupil size (mm)	2.28	0.16
Right pupil size (mm)	2.29	0.16
Number of fixations	12.09	5.85
Duration of fixations (ms)	345.42	141.86
Duration of first fixation (ms)	330.97	178.09
Number of looks to the system	8.56	3.22
Looks duration (ms)	492.22	181.62
Total duration looking at the system (s)	4.07	2.31

**Table 7:** Correlations between measures.

Measure	Usability	RSME	NASA-TLX	Usefulness
**Satisfaction**	ρ = 0.84, *p* < 0.001	ρ = −0.56, *p* = 0.048		
**Pupil Size**		ρ = 0.66, *p* = 0.014		
**Number Fixations**			ρ = 0.57, *p* = 0.042	
**Number of Interface Looks**				ρ = 0.67, *p* = 0.013
